# Modeling Emission Flow Pattern of a Single Cruising Vehicle on Urban Streets with CFD Simulation and Wind Tunnel Validation

**DOI:** 10.3390/ijerph17124557

**Published:** 2020-06-24

**Authors:** Xueqing Shi, Daniel (Jian) Sun, Ying Zhang, Jing Xiong, Zhonghua Zhao

**Affiliations:** 1 State Key Laboratory of Ocean Engineering, School of Naval Architecture, Ocean and Civil Engineering, Shanghai Jiao Tong University, Shanghai 200240, China; shixueqing@sjtu.edu.cn (X.S.); danielsun@sjtu.edu.cn (D.S.); 2 China Institute of Urban Governance, Shanghai Jiao Tong University, Shanghai 200030, China; 3 Shanghai Municipal Engineering Design Institute (Group) Co., Ltd., Shanghai 200092, China; railsj@163.com; 4 Shanghai Institute of Tourism, Shanghai Normal University, Shanghai 200234, China

**Keywords:** traffic emissions, wind tunnel experiment, computational fluid dynamics (CFD), numerical simulation, pollutants

## Abstract

Transportation has become one of the primary sources of urban atmospheric pollutants and it causes severe diseases among city residents. This study focuses on assessing the pollutant dispersion pattern using computational fluid dynamics (CFD) numerical simulation, with the effect and results validated by the results from wind tunnel experiments. First, the wind tunnel experiment was carefully designed to preliminarily assess the flow pattern of vehicle emissions. Next, the spatiotemporal distribution of pollutant concentrations around the motor vehicle was modeled using a CFD numerical simulation. The pollutant concentration contours indicated that the diffusion process of carbon monoxide mainly occurred in the range of 0−2 m above the ground. Meanwhile, to verify the correctness of the CFD simulation, pressure distributions of seven selected points that were perpendicular along the midline of the vehicle surface were obtained from both the wind tunnel experiment and the CFD numerical simulation. The Pearson correlation coefficient between the numerical simulation and the wind tunnel measurement was 0.98, indicating a strong positive correlation. Therefore, the distribution trend of all pressure coefficients in the numerical simulation was considered to be consistent with those from the measurements. The findings of this study could shed light on the concentration distribution of platoon-based vehicles and the future application of CFD simulations to estimate the concentration of pollutants along urban street canyons.

## 1. Introduction

Motor vehicles generally consume fossil fuels and emit harmful pollutants, including carbon dioxide (CO_2_), carbon monoxide (CO), nitrogen oxides (NO_X_), hydrocarbons (HC), and particulates. According to the International Energy Agency, the transportation industry contributes the second largest share of carbon emissions, right after electricity generation from nonrenewable fossil fuels [1]. In large and middle-sized cities, transportation has become the primary source of carbon emissions and air pollution. For example, in the central city of Shanghai, CO and HC emissions from transportation accounted for 92% and 65% of total emission mass into the atmosphere, respectively [2,3]. Moreover, vehicle exhaust from urban traffic is emitted directly into the air near the ground, which is easily inhaled by residents and induces severe diseases [4]. Therefore, it is essential to investigate the generation of exhaust pollutants, and further predict the concentrations of roadside pollutants.

Research on the dispersion of traffic carbon emissions has mainly focused on the spatiotemporal distribution of vehicle exhaust emissions on the surrounding environment after the vehicle exhaust was generated. The focus has been on the pollutant spatiotemporal concentrations, which have mainly been obtained from field measurements, wind tunnel experiments, and operational model or even numerical simulations. Specifically, a wind tunnel experiment is a commonly used experimental method in fluid mechanics, in which one model or object is fixed in an artificial environment (with human-made airflow) to obtain the experimental data in different environments. The operational model is based on numerical solutions of differential equations, which are combined with the semi-empirical formula obtained from observations and wind tunnel simulations, such as the box model, the operational street pollution model (OSPM), and so on [5]. During recent years, computational fluid dynamics (CFD) numerical simulation has been used to model pollutant dispersion in a microscopic environment [6]. The results of a CFD numerical simulation can be compared with corresponding data from field measurements and a wind tunnel experiment. For example, Rivas et al. applied computational fluid dynamics Reynolds-averaged Navier–Stokes (CFD-RANS) simulations to compute the concentrations of NO_2_ and NO_X_ at the pedestrian level of Pamplona city, in 2016, and validated the results through a comparison with measurements provided by fixed and mobile air quality monitoring stations in the city, with a maximum relative error below 30% [7]. Li et al. performed field measurements using instrumented unmanned aerial vehicles (UAVs) with a CFD simulation to investigate pollutant dispersion at a university and to understand the influence of wind fields on PM_2.5_ diffusion patterns [8].

Since the concentration of traffic pollutants is affected by many factors, it is difficult to analyze them simultaneously. Therefore, controlling variables and selecting appropriate input variables have commonly been used in previous studies. Specifically, weather conditions have often been used as the focal points, such as wind velocity, wind directions, temperature, humidity, and so on. Scungio et al. studied the effects of wind speed and street canyon characteristics on traffic-generated particle diffusion through a CFD simulation, after calculating the excess lifetime cancer risk (ELCR), and found that the ELCR became lower with increasing wind speed [9]. Fu et al. studied street canyons in the city center of Beijing and established an urban-scale traffic emission dispersion CFD model [10]. Ultimately, the study pointed out that the canyon geometry had a significant influence on traffic emission concentrations in populated urban areas. Mei et al. investigated the airborne pollutant dispersion in the street canyons under different street length/street width ratios (B/W), and found the street length where with the largest accumulation of pollutants existed and which should be avoided in urban street design [11]. Sun et al. performed CFD simulations to estimate the traffic emission concentrations and found that avenue trees generally increased the pollutant concentration in the canyons, and higher wind velocity intensified the variation tendency of the concentration, rather than changing the fundamental flow patterns [12]. Huang et al. studied the impacts of two tree-planting patterns and eight trunk heights on the emission diffusion in street canyons. [13] The results showed that the concentration of pollutants in an isolated canyon was significantly influenced by trunk heights. Tan et al. constructed four scenarios for different periods, namely morning, noon, afternoon, and late at night, and studied the influence of temperature change on pollutant dispersion [14].

When a motor vehicle passes through streets at a certain speed, it disturbs the ambient atmosphere and the influence on the dispersion of pollutants should be considered. In addition, the exposure concentration felt by pedestrians is often instantaneous, which is more vulnerable to the interference of motor vehicles. Unfortunately, research on traffic produced turbulence (TPT) is somewhat limited. Kastner-Klein et al. conducted wind tunnel experiments to investigate the influence of changing wind field in street canyons, and designed and refined the TPT-related parameters by adapting a vehicle model for a conveyor belt [15]. Similar experimental methods were also used in Khare’s research [16]. However, the research mentioned above identified transport emissions as a continuous and uniform emission source and did not discuss the impact of TPT. Recently, Kim et al. used computational fluid dynamics (CFD) to simulate vehicle induced turbulence in traffic-related pollutant dispersion and first proposed that through superimposing the TPT of a single vehicle in the vehicle fleet, total TPT from multiple vehicles could be estimated [17]. In CFD code ANSYS Fluent, theoretical equations have been provided as available tools for turbulence simulation, such as RANS (Reynolds-averaged Navier–Stokes) [18,19,20,21], LES (large eddy simulations) [22,23,24], GDE (general dynamic equation) [25,26,27], and so on. However, existing studies have seldom made further efforts to explore the specific impact of TPT on emission diffusion. CFD simulation is needed to study the mechanism of TPT affecting pollutant concentration distribution.

This study aimed to assess the emission flow pattern from a single cruising vehicle influenced by TPT using a CFD simulation, which was further validated by carefully designed wind tunnel experiments. First, to understand the generation and diffusion of motor vehicle pollutants, a wind tunnel experiment was designed and calibrated. The pressure distribution of selected points among the vehicle surface was obtained. A flow pattern experiment was also carried out to observe the characteristics of the flow field around the vehicle. Secondly, a CFD numerical simulation was performed to investigate the spatial distribution of pollutant concentrations. The standard k-ε turbulent flow model was chosen for the simulation. After convergence, the pressure coefficients of the same position as the wind tunnel experiment were analyzed. The accuracy of the CFD simulation was confirmed through a comparison between the wind tunnel experiment and the CFD simulation, and therefore the concentration distribution of pollutants around a single motor vehicle could be approximated. The remainder of this paper is organized as follows: A wind tunnel experiment is designed in Section 2 to model a single-vehicle exhaust emitting process; Section 3 presents the CFD numerical simulation details for assessing the pollutant dispersion around a single vehicle, and the results which were compared with those from the wind tunnel experiment for validation purposes; finally, conclusions and recommendations for future work are provided in Section 4.

## 2. Wind Tunnel Experimental Design and Modeling 

A wind tunnel experiment is a classical aerodynamic research tool which involves installing a real (life-size) object or the corresponding scale model within an artificial aero flow field to observe the airflow pattern and interactions with the test object. This study used the wind tunnel equipment from the National Mechanics Experimental Teaching Demonstration Center (NMETDC), Shanghai Jiao Tong University. It is a closed single circumfluence low-velocity wind tunnel, with a circular cross-section, a tube length and a diameter of 1 meter, and a maximum wind speed of about 50 m/s.

### 2.1. Experimental Design

As vehicle type and model can significantly influence automobile aerodynamics characteristics, this study chose a representative vehicle model to generate the testing subject. 

#### 2.1.1. Vehicle Model Selection

According to the ranking statistics of car ownership and passenger car sales released by the China Association of Automobile Manufactures (CAAM), in 2015, the Santana 2000, from Shanghai Automotive Industry Corp., had the highest market share and ranked fifth in basic passenger car sales in China [28]. Considering the classification, sales volume, and market share in China, the Volkswagen Santana 2000 was selected as the representative urban passenger car for the vehicular pollutant distribution modeling in this study.

#### 2.1.2. Testing Model Design and Installation

In this study, a reduced scale model was used, following the rules which imply that the frontal area of the experimental subject should not be more than 5% of the cross-sectional area of wind tunnel testing segment, with the maximum proportion of 7% [29,30]. According to the Santana 2000 selected, the length, width, and height, were 4.5 m, 1.7 m and 1.4 m, respectively, and then a reduced scale of 1:16 was used. As a result, the vehicle model’s frontal area was 87 cm^2^, accounting for 4.43% of the wind tunnel testing segment.

**Figure 1:**
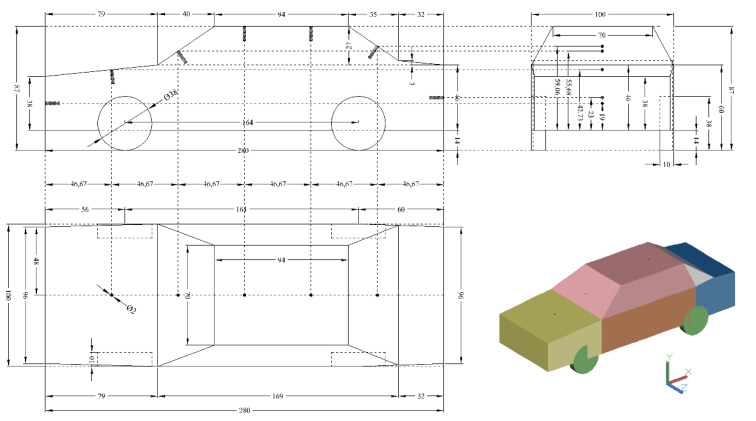
External dimensions of the test model and the pressure measuring points (unit, mm).

According to the selected Santana 2000 model, some detailed accessories, including the outer rear view mirrors, exhaust pipe, sealing strip, and interior decorations were simplified, as presented in Figure 1. The model consisted of six components which included engine cabin (in yellow-green), passenger cabin (in brown), roof (in pink), back window (in white), trunk (in blue), and tires (in green), with three dimensions of 280 × 100 × 87 mm. For the convenience of description, the negative *x*-axis, positive *y*-axis, and positive *z*-axis were defined as the front, upper, and left directions, respectively. 

Since the experimental tunnel had a round cross-section, a baseboard was required to mimic the ground surface, which represented urban roads in reality, thus, reducing the boundary layer generated by the relative movement of airflow. A fixed baseboard in the horizontal plane was used to simulate the surface of roads, with length, width, and thickness settings of 900, 416, and 50 mm, respectively. Polymethyl methacrylate (PMMA) was chosen to manufacture the vehicle model. Accordingly, as the fixed baseboard had comparably low precision requirements and high strength, wood composite board was selected. Finally, the vehicle model was screwed onto the baseboard, with a laser level used to assist the installation, thus, assuring the horizontal plane of the board.

### 2.2. Wind Tunnel Flow Field Calibration

To obtain the potential linear relationship between the fan motor rotation speed and wind velocity, and thus verify the uniformity and stability of the flow field, wind velocities within the tunnel were measured using the German-made Testo 425 Compact Thermal Anemometer, with a wind velocity measurement accuracy of ±0.01 m/s. Considering the height of the testing vehicle model, ten points were chosen to measure the wind velocity as follows: First, the baseboard of the modeled road surface was set as the bench point (1), with the corresponding height defined as 0 mm. Then, another five points were set along the vertical direction, every 20 mm. When the height was above the vehicle model (87 mm), the last four points were selected at the height, every 30 mm, until 220 mm above the bench points. During the experiment, the motor rotation speed was changed from 0 to 350 revolutions per minute (rpm) for every 50 units. For each rotation speed, wind velocities at every point were checked three times, with the mean recorded. Outputs of the wind velocity experiment are presented in Table 1.

Table 1 shows that the wind velocity has an evidently positive correlation with the motor rotation speed, with a Pearson correlation coefficient of 0.99. By choosing the motor rotation speed (*r*) as the independent variable, a linear regression analysis was carried out for the wind velocities of all heights (*v*). The resulting regression function is v=0.096r−1.295, with an adjusted R^2^ of 0.98.

For the same rotation speed, the coefficient of variations (C.V.) of all sampled heights is within 3%, reflecting that the wind velocities of different heights are approximately identical. Meanwhile, at the lower height from 0 to 60 mm, the wind velocities are smaller, which could be caused by the changes of flow field induced by the baseboard. Equations (1)–(3) are introduced to calculate the velocity ratio and average velocity ratio at each height point, and the results are provided in Table 2.

(1) $$\bar{v}\left(r_{j}\right)=\frac{1}{10}\sum_{i=1}^{10}v\left(h_{i},r_{j}\right),$$

(2) $$v^{\prime }\left(h_{i},r_{j}\right)=\frac{v\left(h_{i},r_{j}\right)}{\bar{v}\left(r_{j}\right)}\times 100\%,$$

(3) $$\bar{v}\prime \left(h_{i}\right)=\frac{1}{7}\overset{7}{\underset{j=1}{\sum }}v\prime \left(h_{i},r_{j}\right),$$

where,$\;v\left(h_{i},r_{j}\right)$ is the wind velocity at the point of height $h_{i}$, with the rotation speed $r_{j}$, m/s;$\;\bar{v}\left(r_{j}\right)$ is the average wind velocity of the 10 height points, with the rotation speed $r_{j}$, m/s;$\;v\prime \left(h_{i},r_{j}\right)$ is the wind velocity ratio for giving rotation speed $r_{j}$, at each height point $h_{i}$, %;$\;and\;\bar{v}\prime \left(h_{i}\right)$ is for giving rotation speed $r_{j}$, the average wind velocity ratio of the 10 height points, %.

**Figure 2:**
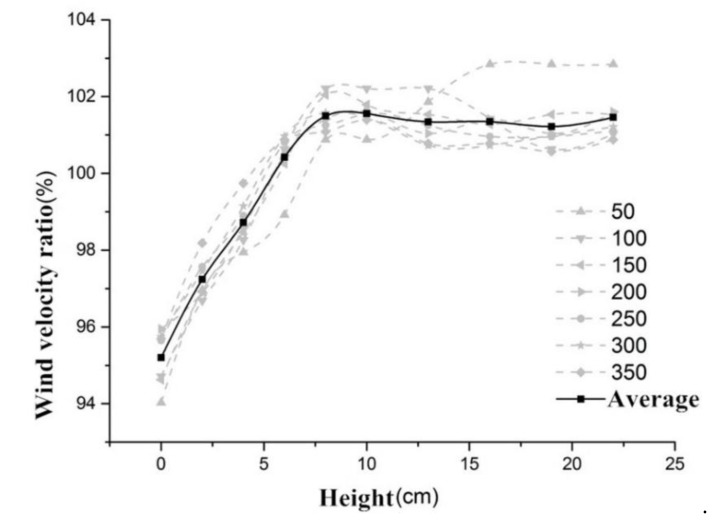
Height and wind velocity ratio (WVR).

By setting the height as the *x*-axis and the wind velocity ratio as the *y*-axis, relationship curves were plotted, as shown in Figure 2. The grey dotted line represents the wind velocity ratio at different rotation speeds, while the solid black line represents the average wind velocity ratio. It was determined that at different rates (50−350 rpm), the trend of wind speed ratio with height was consistent. Large fluctuations were found at rotation speeds of 50 and 100 rpm, which could be induced by the instability of flow field caused by the low wind velocities within the tunnel. The discrepancy of wind velocity ratios is within 0.8% when the height is larger than 60 mm and the motor rotation speed (MRS) is higher than 100 rpm. As a result, the flow field is considered to be stable.

### 2.3. Wind Tunnel Pressure Distribution Experiment

A pressure distribution experiment is a traditional routine test for a wind tunnel investigation, which is particularly important for investigating vehicle aerodynamics characteristics and validating numerical simulations accuracy.

A traditional pressure catheter and liquid column manometer were used to measure the intensity of pressure within the tunnel, with a permissible error of 0.5% for readings. First, the location of the pressure measuring points was determined, as shown in Figure 1. Seven holes were drilled perpendicularly along the midline of the Santana 2000 model, marked by small black cylinders/columns. Both front and rear ends had one hole in the central points, and the remaining five holes were located uniformly along the midline, marked as d1, d2, …, d7 from the front to rear. Each hole had a diameter of 2 mm and was connected to a silicon tube with outer and inner diameters of 2 and 1 mm, respectively.

In this experiment, a standard inclined tube type pressure gauge was adopted, and the conventional liquid column pressure gauge was tilted to increase the sensitivity and reduce the relative error of readings. Specifically, the experimental pressure meter had six adjustable angles (12, 18, 24, 38, 54, and 90 degrees). By setting the inclination angle of the liquid column manometer at degrees of 24, 38, and 54, three groups of experiments were conducted. During each group, the motor rotation speed was increased from 0 to 500, for every 50 units with one measurement. On the basis of Bernoulli’s equation, the pressure coefficient Cp can be calculated using Equations (4)–(7).

(4) $$\Delta \left(r_{j},d_{m}\right)=\left[l\left(r_{j},d_{m}\right)-l_{0}\right]\cdot sin\alpha ,$$

(5) $$\Delta p\left(r_{j},d_{m}\right)=p\left(r_{j},d_{m}\right)-p_{\infty }\left(r_{j},d_{m}\right)=\rho g\Delta h\left(r_{j},d_{m}\right),$$

(6) $$q_{\infty }\left(r_{j},d_{m}\right)=0.5\rho \prime v^{2}\left(r_{j}\right),$$

(7) $$C_{p}\left(r_{j},d_{m}\right)=\frac{\Delta p\left(r_{j},d_{m}\right)}{q_{\infty }\left(r_{j},d_{m}\right)},$$

$\mathrm{where}\;\Delta h\left(r_{j},d_{m}\right)$ is the height difference of liquid column manometer for the particular hole $d_{m}$ at given rotation speed $r_{j}$, m;$\;l\left(r_{j},d_{m}\right)$ is the position/value of liquid column manometer measured from the particular hole $d_{m}$ at given rotation speed $r_{j}$, m;$\;l_{0}$ is the initial position/value of the liquid column manometer, m; α is the inclination angle of liquid column manometer during the measurement, degree;$\;\Delta p\left(r_{j},d_{m}\right)$ is the static pressure difference for the particular hole $d_{m}$ at given rotation speed $r_{j}$, Pa;$\;p\left(r_{j},d_{m}\right)$ is the static pressure measured from the particular hole $d_{m}$ at given rotation speed $r_{j}$, Pa;$\;p_{\infty }\left(r_{j},d_{m}\right)$ is the inflow static pressure measured from the particular hole $d_{m}$ at given rotation speed $r_{j}$, Pa; ρ is the density of the liquid used in the manometer, set as 1000 kg/m^3^ for water used in this study; g is the acceleration of gravity, set as 9.8 kg/m^2^ in this study;$\;q_{\infty }\left(r_{j},d_{m}\right)$ is the inflow dynamic pressure measured from the particular hole $d_{m}$ at given rotation speed $r_{j}$, Pa; ρ′ is the air density, which is chosen as 1.22 kg/m^3^ for the temperature of 16 °C, and atmospheric pressure of 101.3 kPa measured during the experiment;$\;v\left(r_{j}\right)$ is the controlled wind velocity corresponding with rotation speed $r_{j}$, calculated by the regression equation $v\left(r_{j}\right)=0.096r_{j}-1.295$, m/s;$\;\mathrm{and}\;C_{p}\left(r_{j},d_{m}\right)$ is the pressure coefficient $C_{p}$ calculated for the particular hole $d_{m}$ at given rotation speed $r_{j}$.

**Figure 3:**
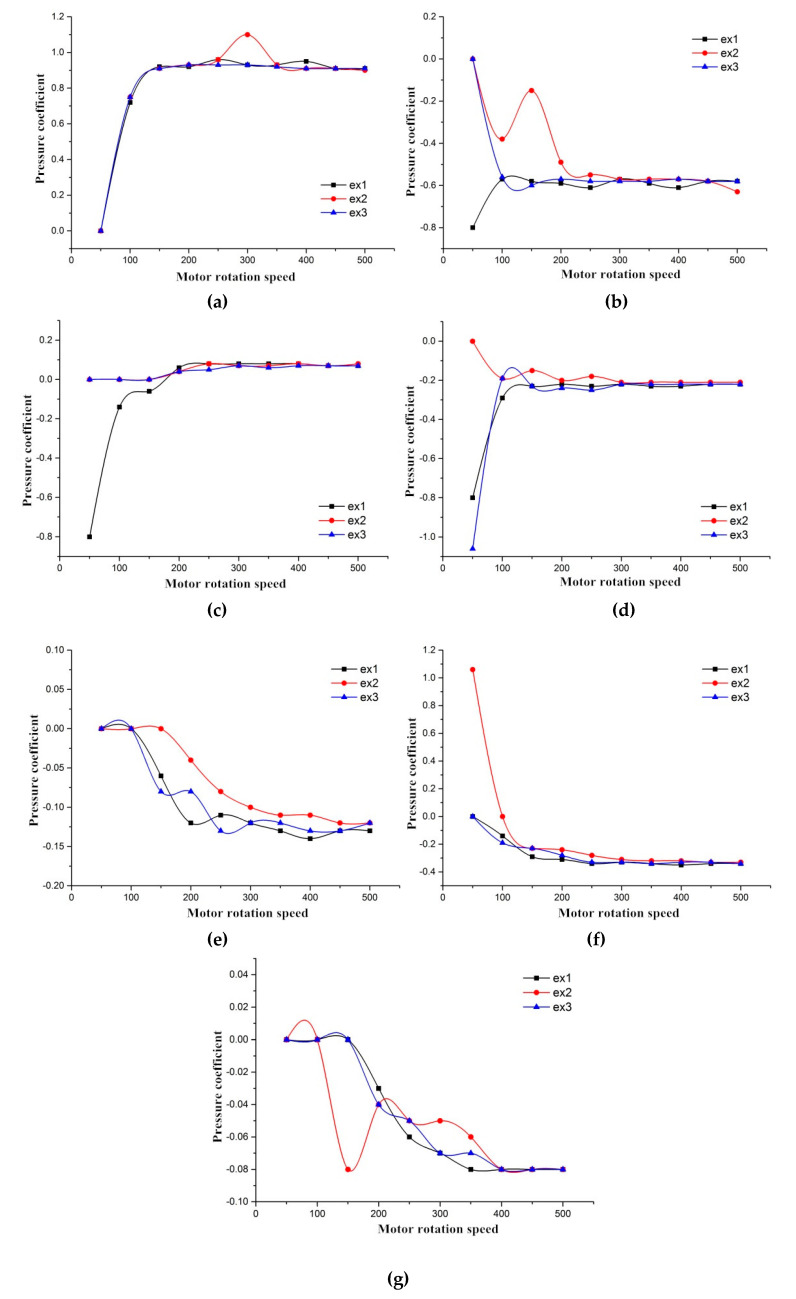
Motor rotation speed (MRS) and pressure coefficient. (**a**) Pressure measuring hole d_1_; (**b**) Pressure measuring hole d_2_; (**c**) Pressure measuring hole d_3_; (**d**) Pressure measuring hole d_4_; (**e**) Pressure measuring hole d_5_; (**f**) Pressure measuring hole d_6_; and (**g**) Pressure measuring hole d_7_.

By plotting the motor rotation speed on the *x*-axis and the pressure coefficient on the *y*-axis, the scatter plot with trend lines added for each measuring hole was plotted and is shown in Figure 3a–g. The pressure coefficient for different rotation speeds are plotted, and the three colorful solid lines (black, red, and blue) correspond to the three measurements within the experiment.

Figure 3 shows that for all seven holes, changes or trends of pressure coefficient are consistent with motor rotation speeds. The pressure coefficient demonstrated certain instability during the low rotation speeds and converged to given values with rotation speed increases, possibly, due to the following reasons: (1) From the experimental results of field calibration, we found that the flow field within the experimental tunnel segment was unstable under low motor rotation speeds, which could further induce the instability of the pressure coefficient and (2) under low motor rotation speeds, the Reynolds number (Re) was too small to simulate the real-world conditions experienced by vehicles. The so-called Reynolds number is a dimensionless number that can be used to characterize the flow of fluid, and it can be calculated by Equation (8). The pressure coefficients for all holes become stable, when the rotation speed is larger than 400 rpm (as observed from Figure 3), which is approximated as the wind velocity around 37.1 m/s, based on the obtained regression function (v=0.096r−1.295). The Reynolds number independent test is carried to guarantee the reliability of the model for the comparison between the wind tunnel models and the real scale model. The corresponding Re of the wind tunnel model is calculated to be 0.7×10^6^. The result is consistent with the conclusion from Tilch et al. [31], where Re was required to be larger than 0.6 × 10^6^ for a qualified wind tunnel experiment. By the similarity analysis method, the Reynolds number (Re) was used as the reference parameter, and the actual inflow speed of the flow field around vehicles corresponding to the flow field in the wind tunnel was calculated to be 8.31 km/h.

(8) Re=ρVL/μ,

where  Re is the Reynolds number, a dimensionless number. For fluid density, this paper uses the air density at a temperature of 16 °C and pressure 101.325 kPa, equaling to 1.22 kg/m^3^. *V* is the velocity character of the flow field. For the outflow problem, V generally takes the forward inflow velocity, and here, it uses the wind tunnel velocity corresponding to the chosen rotation speed (400 rpm), which is 37.1 m/s. *L* is the characteristic length. For the outflow problem, L generally takes the main dimensions of objects, the Santana 2000 model vehicle length is 0.28 m. Here, the dynamic coefficient of viscosity is $18.023\times 10^{-6}\;N\cdot s/m^{2}$.

### 2.4. Wind Tunnel Flow Pattern Experiment

A flow pattern experiment is an important supplement to a pressure distribution experiment during wind tunnel experiments. It obtains parameters, such as flow speed at fixed points within the field, and then analyzes the characteristics of the flow field. The main experimental methods include laser doppler velocimetry (LDV), particle image velocimetry (PIV), and so on. In addition, the experiment focuses on field observation, in which ribbon method, oil film method, and smoke flow method are frequently used. In this study, the smoke flow method was chosen to observe the pollutant dispersion within the vehicle rear area. With this method, the flow pattern of fluid around the model is shown through the smoke emitted from a smoke generator.

The YWQ-701 aerosol generator (from Qingdao Linding Aerosol Generator Technology Co. Ltd., Qingdao, China) was selected for the flow pattern experiment. The blow head of the aerosol generator was connected with a silicon tube, with an outer diameter of 2 mm, which was fixed at the right rear position to mimic the exhaust pipe. The flow rate of the aerosol generated was 2000 cubic feet per minute.

**Figure 4:**
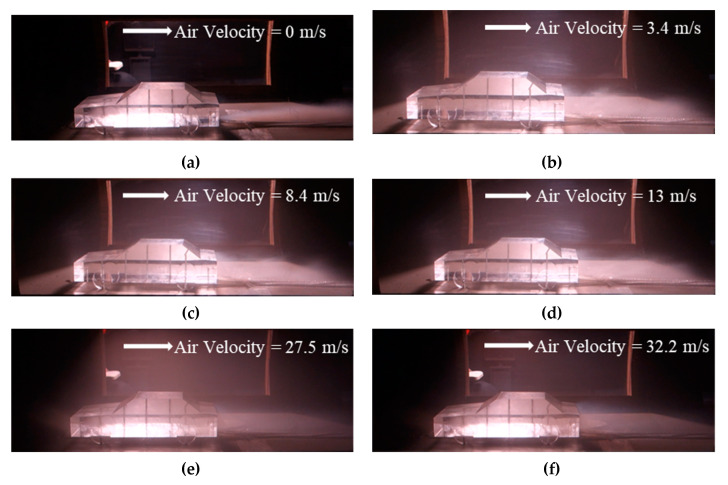
Typical flow patterns, (**a**) Flow Pattern 1; (**b**) Flow Pattern 2; (**c**) Flow Pattern 3; (**d**) Flow Pattern 4; (**e**) Flow Pattern 5; (**f**) Flow Pattern 6.

Typical flow patterns for vehicle emissions were captured (Figure 4). First, Flow Pattern 1, as presented in Figure 4a, mimics the initial situation, in which the motor did not rotate and the wind velocity was 0 m/s. Jet streamline smoke is evidently generated in the rear part of the vehicle. Flow Patterns 2 to 4 (Figure 4b–d) reflect an observable group of circulating flow regime under low wind velocities, whereas Flow Patterns 5 and 6 (Figure 4e–f) reflect an observable group of circulating flow regime under high wind velocities. For each circulating flow regime group, under and after the vehicle rear cover, and within the width of the vehicle, high concentration areas were identified, which were also with significant fluctuations.

From Figure 4, fluctuations in flow pattern were observed. For example, complex turbulence was identified around the rear part of vehicle, because of the interactions of separated flows from all directions around the vehicle. This was consistent with existing research on the rear streamline of the hatchback passenger car [32] which, to a certain extent, suggested the wind tunnel experiment could simulate the actual flow field of the vehicle in a certain precision range based on the careful experimental design.

## 3. Assessing the Airflow Distribution Using CFD Simulation

In addition to the wind tunnel experiment, a CFD-based numerical simulation was carried out to investigate the distribution of the pollutants around a single vehicle in a flow field. The microscopic single vehicle wind tunnel experiment provides calibration and a validation platform for the CFD-based numerical simulation. Results and findings from the wind tunnel experiment can be further used to improve the efficiency of CFD simulation, and thus to prove methodological feasibility for the CFD-based urban transportation emission simulation [33].

### 3.1. Assumptions

Automobile numerical simulation has been used for investigating system aerodynamics in certain engineering areas, such as mechanical engineering and automotive engineering. Nevertheless, for traffic emission studies, the required modeling accuracy is generally not as high as the aerodynamics models. As a result, the following assumptions were made for model simplification purposes:

**Assumption** **1.** *On the basis of the theory of hydrodynamics, both air and vehicle emissions are considered to be viscous and non-compressible fluid.*

**Assumption** **2.** *The vehicle model is considered to be a closed entity, the possible inner and outer flow field exchange by sunroof, side window, or external circulation system is ignored.*

**Assumption** **3.** *The entire outer flow field is not affected by the detail components or small shape changes of the vehicle. The simulation model is the same as the one used in the wind tunnel experiment shown in Figure 1.*

**Assumption** **4.** *For further handling and analysis, the numerical simulation used steady-state calculation under natural wind for vehicle pollutants dispersion based on the same model in the wind tunnel experiment.*

### 3.2. Computational Domain

The major adjustments for the CFD simulation as compared with the wind tunnel model included the following: 

1. Constructed was based on the real-life scale, with a reduced scale of 1:1;

2. The vehicle tires were simplified from cylinder to cuboid, with the length similar to the diameter of the real-life tire, and the height as the chassis of the vehicle to the ground;

3. The exhaust pipe was modeled as a cylinder with a radius of 4 cm, which was attached to the chassis, and 0.45 m to the centerline of the vehicle body.

On the basis of the simulation model, the three dimensions of computational domain were set as 48.48 m, 11.6 m, and 7 m, respectively. To ensure the complete divergence of the flow field within the domain, the vehicle model was glued to the bottom surface, with 14 and 30 m in front and back, and 5 m on both left and right sides. During the configuration, to ensure the continuity of domain and avoid repeat surface or cavities, the overlapped surfaces between the tires and main vehicle body, or the tires and the bottom surface were removed.

The ANSYS ICEM 16.0 software package (ANSYS, Inc., Canonsburg, United States) was used to create the mesh file for the CFD simulation, through which the CFD model was discretized as a computational model composed of various elements to conduct finite element calculation [34]. In this paper, the decided mesh number was 1.61 million, and the maximum global size of the mesh was set as 256 mm^2^, based on recommendations from previous studies [35].

### 3.3. Boundary Conditions

The FLUENT 16.0 software package (ANSYS, Inc., Canonsburg, PA, USA) was introduced for the numerical simulation, with a standard k-ε turbulent flow model used to replicate the airflow within the studying area. For the CFD simulation, the four types of boundary conditions included velocity inlet, pressure outlet, symmetry, and wall. The corresponding configurations are described in the follows subsections.

#### 3.3.1. Velocity Inlet

During the CFD simulation, the front surface of the domain was set as velocity inlet, and the inflow wind velocity was set as 2.5 m/s, similar to the field situation, and perpendicular to the velocity inlet. However, the value was set at 40 m/s during the wind tunnel experiment, as a reduced scale of 1:16 was used for the Santana 2000 within the wind tunnel experiment. Moreover, values of the turbulence parameters for the flow field boundaries were defined based on turbulence intensity and hydraulic diameter. Intensity for the fully developed core turbulence is estimated by Equation (9), while the hydraulic diameter is calculated using Equation (10) as follows:

(9) $$I=0.16{Re}^{-1/8}=2.71\times 10^{-2},$$

(10) $$D_{H}=4S/C=8.7m,$$

where  I is the turbulence intensity, where Re can be estimated from the parameters, including air density and atmospheric pressure, and so on;$\;D_{H}$ is the hydraulic diameter, m; *S* is the circulation area for the boundary of velocity inlet, m^2^; and *C* is the wetted perimeter for the boundary of velocity inlet, m.

In addition to setting the front surface of the domain as the velocity inlet, the rear exhaust pipe surface was also set as the velocity inlet. On the basis of Assumption 1, both air and vehicle emissions were considered viscous, non-compressible, and the emission inflow velocity, perpendicular to the velocity inlet, can be approximated by Equation (11) as follows:

(11) $$v_{CO}=\frac{\left(f\cdot v\right)}{\left(\rho_{CO}\cdot S\right)}=2.83\times 10^{-3}m/s,$$

where $v_{CO}$ is the emission inflow speed, m/s; f is the standard emission factor, in China Stage IV, defined as 0.68 g/km for a small passenger car driving on typical urban roads (30 km/h) [36]; v is the vehicle driving speed, set as 30 km/h for driving on typical urban roads;$\;\rho_{CO}$ is the density, set as 1.25 g/L for CO; and S is the cross-section area for vehicle exhaust pipe, set as $1.6\times 10^{-3}$ m^2^.

Values of the turbulence parameters for the flow field boundary can also be calculated by Equations (4) and (5), as the turbulence intensity and the hydraulic diameter were obtained as $6.94\times 10^{-2}$ and 4.5 cm, respectively.

#### 3.3.2. Pressure Outlet 

The rear surface of the domain is set as the pressure outlet, and the direction of the outlet flow is normal to boundary.

#### 3.3.3. Wall 

In the viscous flow, the default wall condition is a no-slip wall. In this study, the vehicle body and the bottom surface of the computational domain are set as the wall condition. 

#### 3.3.4. Symmetry

During the steady state, the tailpipe is not in use, and consequently, the flow fields around the vehicle body, including the left side, top, and right side, are symmetrical, and can be set as symmetry conditions.

### 3.4. Simulation and Validation

**Figure 5:**
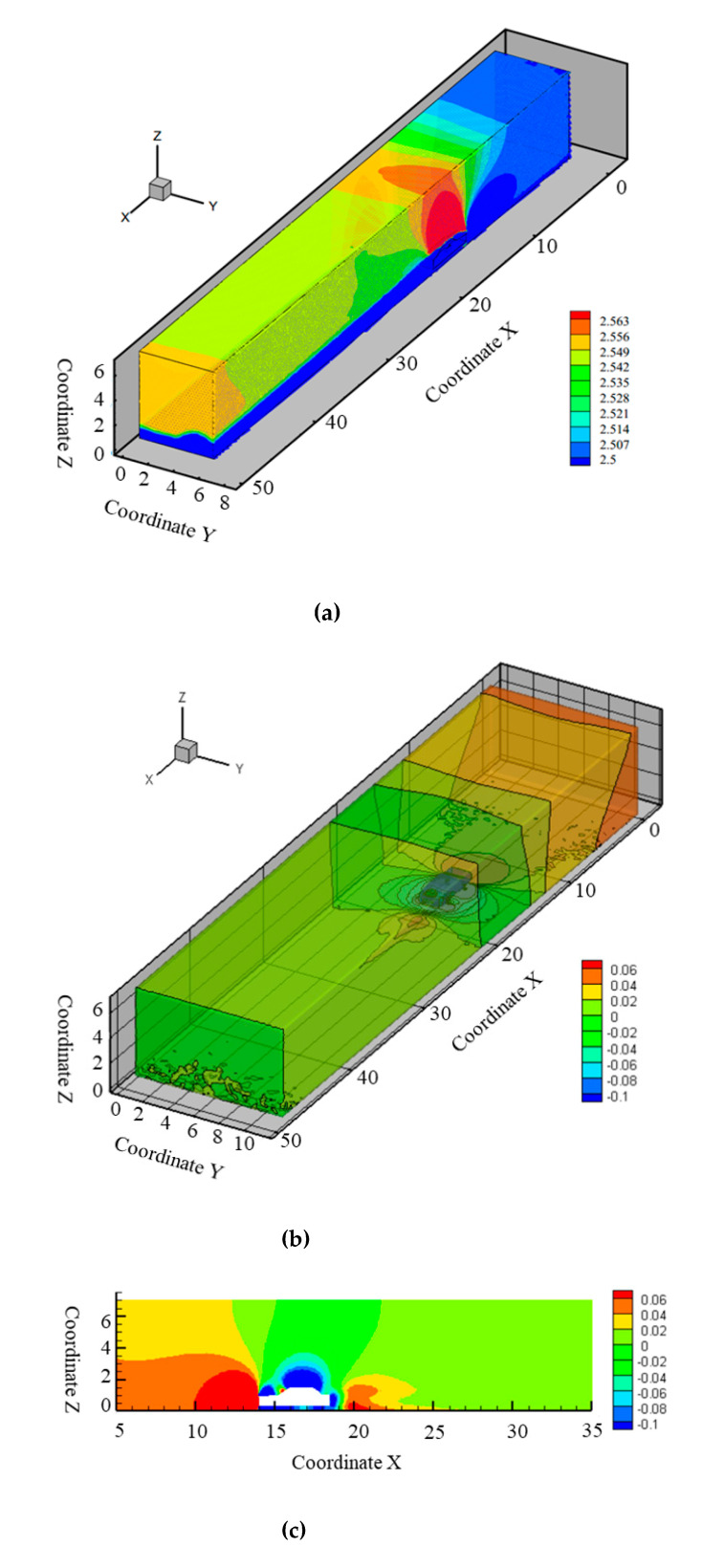
Results of computational fluid dynamics (CFD) simulation and validation. (**a**) Velocity contour of steady-state simulation for a single vehicle; (**b**) Pressure coefficient contour of steady-state simulation for a single vehicle; (**c**) Pressure coefficient contour of the center surface.

After the steady-state calculation, the results, including the pressure coefficient and the velocity of flow field, etc., were imported into Tecplot 360. The velocity field and pressure coefficient were obtained and compared with the results from the wind tunnel experiment, as presented in Figure 5.

#### 3.4.1. Velocity Field

By cutting the calculation domain along the midline of the vehicle body, the velocity contour for the left side is presented in Figure 5a, while the right side is symmetrical. It can be figured out that the fields of three regions, located in the upper front, the upper above, and upper rear of the vehicle, have higher velocities, while the space below the vehicle body has a stable/constant velocity.

#### 3.4.2. Pressure Coefficient

Figure 5b presents the pressure coefficient contour of steady state within the calculation domain, whereas Figure 5c presents the pressure coefficient contour along the center cross-section. As shown, the following trends were identified for the pressure coefficient contour of the numerical simulation:

- Higher positive pressure was formed within the vehicle head frontal area;
- Negative pressure was formed right above the vehicle head, while positive pressure was formed above the windshield; and
- Lower negative pressure was formed above the vehicle roof, while negative pressure was formed after the rear part of vehicle.

**Figure 6:**
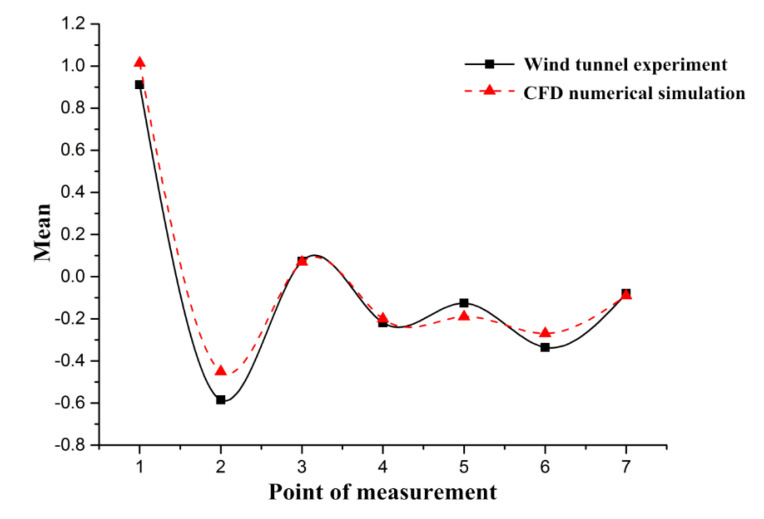
Pressure coefficient comparison.

To compare the results of the pressure distribution experiment from the wind tunnel experiment with those from the CFD simulation, seven points were chosen right at the same position along the midline of vehicle, as in the wind tunnel experiment. The pressure coefficients were measured and compared with those from the wind tunnel experiment, as presented in Figure 6. For measuring points 1, 3, 4, and 7, the pressure coefficients from the numerical simulation are consistent with the results from the wind tunnel experiment, with the error below 10%. For the measuring points 2 and 6, results from the numerical simulation are higher, while the value from point 5 is smaller, all with the error between 20% and 30%. The main possible reasons for the discrepancy could relate to the three-dimensional mesh file division for the CFD simulations, in which the regular tetrahedron and hexahedron elements were mixed within a three-dimensional area. However, it can be concluded that the distribution trend of all pressure coefficients in the numerical simulation was rather similar and consistent with the pressure coefficients from the wind tunnel experiment, since the Pearson correlation coefficient between the numerical simulation and the wind tunnel measurements was 0.98, indicating a strong positive correlation. This further shows that the CFD model can simulate real flow fields in a wind tunnel and have high precision. Consequently, the CFD numerical simulation model used in this investigation was considered to replicate reality effectively.

**Figure 7:**
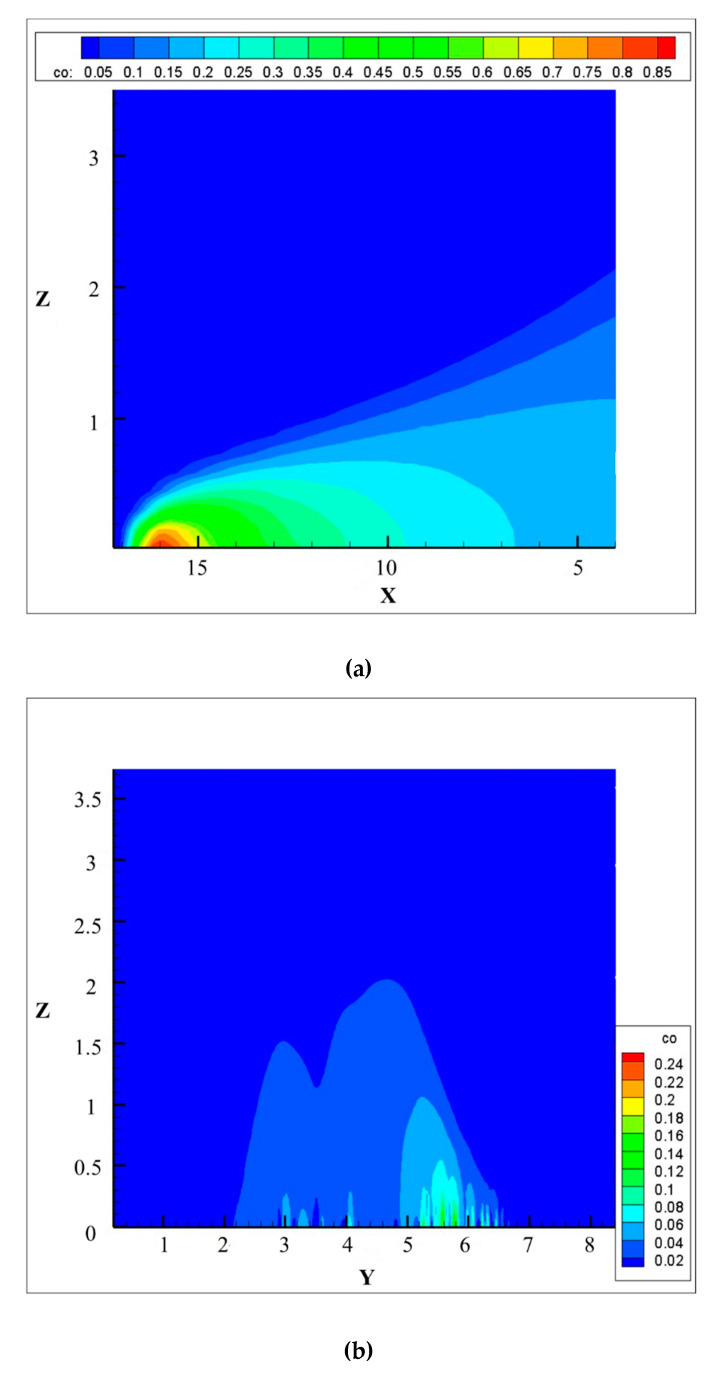
Contour map of carbon monoxide mass fraction at cutting surface. (**a**) Vertical section along the axis of the vehicle model; (**b**) Cross-section along the *Y*-axis (X = 10 m).

After verifying the reliability of the CFD simulation, the CO concentration distribution around the vehicle can be analyzed. Due to the large size of the CFD simulation model and low emission intensity of carbon monoxide source, the information provided by the mass fraction distribution contour of the CO in the whole flow field is limited. Therefore, it is more appropriate to select the representative section for analysis. The longitudinal section of the vehicle along the *X*-axis is shown in Figure 7a, and the cross-section along the *Y*-axis (X = 10 m) is shown in Figure 7b. The color close to red indicates that the mass fraction of CO and the concentration of pollutants are high, while blue indicates that the concentration is low. It can be seen from the figure that the diffusion process of carbon monoxide mainly occurs in the range of 0−2 m above the ground. The closer to the emission source, the higher the concentration of carbon monoxide. In the environment of no buildings around the road, there is no obvious carbon monoxide accumulation area on the roadside.

## 4. Conclusions

This study investigated traffic pollutant concentration dispersion from a single motor vehicle using wind tunnel experiments and a CFD simulation. A typical passenger car model, Santana 2000, was chosen to analyze traffic emission dispersion under vehicle disturbance. A CFD software package was used to set up a numerical simulation model, with the results compared and validated by the wind tunnel experiment. The distribution of pollutants around the vehicle indicated that the concentration of pollutants around the vehicle exhaust pipe was relatively high, and the carbon monoxide diffusion was concentrated within a height of two meters above the ground. The main contribution includes combining the wind tunnel experiment with the CFD tools, thus, investigating traffic emission dispersion around a single motor vehicle from a microscopic perspective, which could further improve the accuracy of traffic emission forecasting. The results can provide technical ways to investigate traffic emission distribution along representative urban roads for urban planners, as well as architectural designers [37,38]. 

While the results are promising, since research on using the synthetic approach of wind tunnel experiments and CFD simulation to study traffic emission dispersion is, by and large, relatively less common, limitations are unavoidable. For the simplified model of platooned traffic flow, we used a single small-size vehicle in the wind tunnel experiment and the CFD simulation for comparison due to the volume limit of the wind tunnel test section. An advantage of using a single vehicle model it that it lays a foundation and demonstrates the laws for calculation of a complex fleet model. However, platooned traffic flow contains interactions among automobile outflow fields in car-following situations, which needs further studying. For the vehicle line design difference between Santana 2000 models and new version vehicle models, we believe such a transformation would not bring an obvious bias to the final results. For the model applied, the results for the wind tunnel experiment and the CFD simulation show consistent trends (Figure 6). Although the pressure coefficient of the curve could vary slightly as the vehicle is updated, the consistency would not change. For the simplified exhaust emission in the wind tunnel experiment, despite the merits of the wind tunnel experiment, it also brings some problems to solve. The wind tunnel experiment, as an important method to validate the CFD simulation results, has no capability of establishing an actual exhaust emission environment, while the CFD simulation software can handle it by using parameter and boundary condition settings. Hot tailpipes and complicated chemical components of emissions are difficult to simulate in wind tunnel, which could result in a slight difference between the wind tunnel experiment and the CFD simulation results, especially the higher error in points 2, 5, and 6 in Figure 6.

Further studies need to be conducted to improve the performance of the model. First, additional vehicle models could be considered. Secondly, field data collection is one significant component in traffic emissions modeling and is especially important. In this research, a wind tunnel experiment and a CFD simulation were used to assess the emissions around the vehicle. As a follow-up study, the field vehicle engine dynamometer test could be used to assess real-life emissions around the vehicle under certain weather and topographic situations. Lastly, this study provides a promising direction for transportation environmental studies. However, the results have to be extended to investigate emissions from a vehicle platoon and the distribution in urban street canyons, which could form a very important extension to the current research.

## Figures and Tables

**Table 1:** Motor rotation speed (MRS) and wind velocity (unit, m/s).

MRS (rpm)	Height (cm)										Mean	C.V.
	0	2	4	6	8	10	13	16	19	22		
50	3.2	3.3	3.3	3.4	3.4	3.4	3.5	3.5	3.5	3.5	3.4	0.03
100	8.0	8.2	8.3	8.5	8.6	8.6	8.6	8.6	8.5	8.5	8.4	0.03
150	12.3	12.6	12.8	13.1	13.3	13.3	13.2	13.2	13.2	13.2	13.0	0.03
200	17.0	17.1	17.4	17.8	17.9	18.0	17.9	17.9	17.9	18.0	17.7	0.02
250	21.6	22.0	22.3	22.8	22.9	22.9	22.9	22.8	22.8	22.8	22.6	0.02
300	26.3	26.8	27.2	27.7	27.9	27.9	27.7	27.7	27.7	27.8	27.5	0.02
350	30.8	31.6	32.1	32.4	32.5	32.6	32.4	32.4	32.3	32.4	32.2	0.02

**Table 2:** Wind velocity ratio (WVR) and average velocity ratio (AVR) under different motor rotation speeds.

Height (cm)	Wind Velocity Ratio (WVR), %							AVR, %
	rpm = 50	rpm = 100	rpm = 150	rpm = 200	rpm = 250	rpm = 300	rpm = 350	
0	94.0	94.7	94.6	95.9	95.6	95.8	95.7	95.2
2	97.0	96.7	96.9	96.9	97.6	97.5	98.2	97.2
4	97.9	98.3	98.5	98.6	98.9	99.2	99.7	98.7
6	98.9	100.6	100.3	100.5	100.8	101.0	100.9	100.4
8	100.9	102.2	102.0	101.4	101.3	101.6	101.1	101.5
10	100.9	102.2	101.8	101.6	101.5	101.5	101.4	101.6
13	101.9	102.2	101.5	101.0	101.3	100.7	100.8	101.3
16	102.8	101.4	101.3	101.4	101.0	100.7	100.8	101.3
19	102.8	100.6	101.5	101.0	101.0	101.0	100.6	101.2
22	102.8	101.0	101.5	101.6	101.1	101.2	100.9	101.5
